# Calorimetric Monitoring of the Sub-T_g_ Crystal Growth in Molecular Glasses: The Case of Amorphous Nifedipine

**DOI:** 10.3390/molecules30081679

**Published:** 2025-04-09

**Authors:** Roman Svoboda

**Affiliations:** Department of Physical Chemistry, Faculty of Chemical Technology, University of Pardubice, nam. Cs Legii 565, 532 10 Pardubice, Czech Republic; roman.svoboda@upce.cz

**Keywords:** amorphous nifedipine, GC growth, Raman microscopy, DSC, kinetic prediction

## Abstract

Non-isothermal differential scanning calorimetry (DSC) and Raman microscopy were used to study the crystallization behavior of the 20–50 μm amorphous nifedipine (NIF) powder. In particular, the study was focused on the diffusionless glass-crystal (GC) growth mode occurring below the glass transition temperature (T_g_). The exothermic signal associated with the GC growth was indeed directly and reproducibly recorded at heating rates q^+^ ≤ 0.5 °C·min^−1^. During the GC growth, the α_p_ polymorphic phase was exclusively formed, as confirmed via Raman microscopy. In addition to the freshly prepared NIF samples, the crystallization of the powders annealed for 7 h at 20 °C was also monitored—approx. 50–60% crystallinity was achieved. For the annealed NIF powders, the confocal Raman microscopy verified a proportional absence of the crystalline phase on the sample surface (indicating its dominant formation along the internal micro-cracks, which is characteristic of the GC growth). All DSC data were modeled in terms of the solid-state kinetic equation paired with the autocatalytic model; the kinetic complexity was described via reaction mechanism based on the overlap of 3–4 independent processes. The kinetic trends associated with decreasing q^+^ were identified, confirming the temperature-dependent kinetic behavior, and used to calculate a theoretical kinetic prediction conformable to the experimentally performed 7 h annealing at 20 °C. The theoretical model slightly underestimated the true extent of the GC growth, predicting the crystallinity to be 35–40% after 7 h (such accuracy is still extremely good in comparison with the standard kinetic approaches nowadays). Further research in the field of kinetic analysis should thus focus on the methodological ways of increasing the accuracy of considerably extrapolated kinetic predictions.

## 1. Introduction

Amorphous drugs play a crucial role in modern pharmaceutical development [1,2,3,4,5,6,7]. They are particularly relevant in the case of poorly water-soluble drugs (BCS classification II and IV [8,9,10,11]), where the disordered structure of the glassy/amorphous state results in higher free energy, higher reactivity, and enhanced apparent solubility [12,13,14,15]. The faster dissolution then translates into better absorption of the active pharmaceutical ingredients (APIs) in the gastrointestinal tract and, thus, higher bioavailability [16,17,18]. Consequently, amorphous drugs can be administered in lower doses while achieving the same therapeutic effect as their crystalline counterparts, effectively reducing side effects and increasing patient compliance (associated with fewer pills or less frequent dosing). In addition, amorphous drugs are more versatile with regard to both their incorporation into various drug delivery systems and formulation outside of the patent legislation (which is strictly limited for the API’s crystalline forms) [19,20,21]. The crystalline-to-amorphous conversion also offers an opportunity for rescuing/repurposing certain promising API candidates that were omitted from clinical trials due to poor bioavailability [22,23,24].

Apart from these benefits, the amorphous APIs also bear one major disadvantage, namely their thermodynamic instability [25,26]. Due to its higher Gibbs free energy, the amorphous state will always have the tendency to spontaneously transform into the crystalline state (this is universally valid for all temperatures below the melting point *T_m_*). For ordinary glasses, their thermal stability is relatively high below their glass transition temperature (*T_g_*), where the high viscosity (usually *η* ≥ 10^12^ Pa·s) stabilizes the material kinetically [27,28]. This is, however, not the case for the low-molecular glasses, where several specific crystallization mechanisms come into play. The main mechanism is the standard volume-located crystal growth from nuclei homogeneously dispersed in the bulk glass. The most well-known crystal growth mechanism associated with amorphous drugs is the diffusionless glass-crystal (GC) growth [29,30,31], which represents a rapid increase in the growth rate below *T_g_*. The growth proceeds along micro-cracks and is ceased at *T* > *T_g_* due to the increased molecular mobility hindering the propagation of the growth front. The second well-known growth mechanism is enhanced surface growth [32,33], where the key role plays markedly higher surface mobility (self-diffusion) due to the lower coordination and packing constraints. Other types of crystal growth include: (1) mobility-induced crystallization in localized regions (nanocrystallization in soft spots or transiently mobile regions related to spatially heterogeneous dynamics in the glassy matrix); (2) the diffusion-limited growth from seeded crystalline phase (where the energy barrier is significantly lower compared to the combined nucleation and growth processes) [34,35,36,37].

These crystal growth modes represent a large hindrance to the potentially routine future exploitation of the amorphous APIs in drug development. Since many commonly used APIs exhibit *T_g_* near the laboratory temperature, the long-term storage and/or processing of such amorphous APIs can lead to the uncontrolled partial devitrification (crystallization) of the amorphous phase. This may have serious consequences for the patient’s health, as the decreased solubility and bioavailability can result in dosage inconsistencies, subtherapeutic effects, or treatment failure [38,39,40,41]. For this reason, the detailed knowledge of these crystal growth mechanisms and their kinetics is of critical importance for the reliable and controlled utilization of amorphous APIs in modern medicine.

From the pharmaceutical practice point of view, the GC growth and the enhanced surface growth modes are the most important. They can occur spontaneously even below *T_g_*, representing a threat to the physico-chemical and thermal stability of the amorphous APIs [24,25,26,29,30,31,32,33,34,35,36]. These growth modes are particularly relevant for the class II and III APIs [25] with *T_g_* below ~50 °C, for which these growth modes can occur during the manipulation, processing, or storage at laboratory temperature. Nowadays, the GC growth kinetics is ordinarily studied by means of optical microscopy, where a thin film of the molten API is usually quenched between two glass slides (microscopy coverslips), and the crystal growth front is studied for either the sandwiched film or the film with the upper coverslip removed [29,30,32]. However, such conditions are far from what is usually encountered during pharmaceutical practice, where amorphous APIs are used/processed in the form of powders. Since the spectroscopic techniques (Raman or infrared) do not usually homogeneously penetrate the volume of the larger powder grains (and the collected signal does not have to be proportional to the monitored sample volume), and the in situ X-ray diffraction is difficult to realize without a strong synchrotron source, there is no reliable and accessible experimental technique for direct monitoring of the GC growth in powdered amorphous APIs. It has been only recently shown [42,43,44] that the GC growth can be directly monitored calorimetrically using a non-isothermally operated differential scanning calorimeter (DSC). Under certain conditions, i.e., at low heating rates *q^+^* and/or for a nucleated sample (depending on the API), the sub-*T_g_* crystal growth manifests itself as an isolated exothermic crystallization peak. The goal of the present paper is to systematically explore the reliability, accuracy, and reproducibility of such DSC measurements. Note that all these aspects are particularly important in the case of the crystal growth in amorphous APIs, where several overlapping growth mechanisms can occur in dependence on even small changes in experimental conditions. In addition, the methodology for evaluating the macroscopic GC growth kinetics will be developed and tested based on different thermal histories of the amorphous material.

In the present study, finely powdered amorphous nifedipine (NIF) will be used as a model material. Nifedipine is a poorly water-soluble (<5 μg·mL^−1^ [45]) calcium channel blocker that is commonly employed in obstetrics as a tocolytic agent to delay preterm labor [46] or in the management of hypertensive disorders in pregnancy [47]. Its vasodilatory effect is beneficial in conditions like hypertension, angina, and Raynaud’s disease [48]. The microscopic studies of its GC growth [49,50,51,52,53] have indeed shown that all polymorphic forms of NIF exhibit an increase in the crystal growth rate (by at least one order of magnitude) below *T_g_*. However, as mentioned above, the microscopy investigation was performed only for amorphous layers formed between two coverslips. Here, the “bulk” growth rate is measured with both coverslips still attached to the amorphous layer (the growth front propagates throughout the whole layer thickness), and the surface growth rate is measured with the upper coverslip removed. Nevertheless, both these approaches are rather specific regarding the growth conditions—the “bulk” growth rate is influenced by the layer/substrate interface (and thus does not have to represent the true growth rate within a larger bulk ingot of grain); the surface growth then corresponds to the growth influenced by the surface defects and micro-cracks that form during the removal of the coverslip (note that this is a convenient necessity because, ordinarily, the crystal growth in low-molecular glasses initiates only from such defects and practically never from a smooth surface). Hence, in the present paper, the aim is to study the sub-*T_g_* crystal growth for the finely powdered amorphous material (NIF), i.e., for the material in the form very close to that actually utilized in the pharmaceutical practice.

## 2. Results

**Figure 1:**
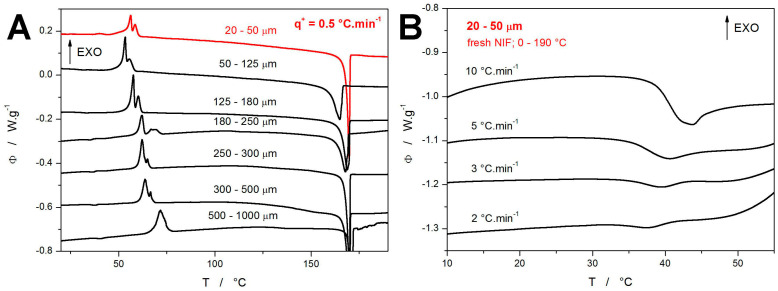
(**A**) DSC curve obtained at 0.5 °C·min^−1^ for the freshly prepared NIF powder with particle size 20–50 μm (red data, present research) compared with the DSC curves obtained under similar conditions for NIF powders with larger particle sizes (black data, taken from [44]). (**B**) DSC curves obtained for the freshly prepared 20–50 μm NIF powder at different *q^+^*; the graph is zoomed-in on the glass transition region. Exothermic effects evolve in the upward direction in both graphs.

In the default series of the DSC measurements, the freshly prepared NIF powder with particle sizes in the 20–50 μm range was subject to the heating scans performed in the 0–190 °C range at different heating rates *q^+^*. The DSC curve obtained at *q^+^* = 0.5 °C·min^−1^ is shown in Figure 1A, together with similar measurements (published in [44]) obtained for coarser NIF powders. In contrast to the crystallization double-peaks (exothermic signals in the ~50–75 °C range) obtained for the coarser NIF powders, the DSC curve corresponding to the present 20–50 μm powder exhibits not only a prolonged onset tail of the double-peak but also an additional (preceding) small exothermic peak at ~40 °C, which roughly corresponds to the onset of the glass transition effect manifesting in the case of the coarser powders. As will be shown later, this small crystallization peak represents the combined GC growth and enhanced surface growth mechanisms. In this regard, it is clear that this occurrence of the small crystallization peak requires a very high concentration of the micro-cracks and internal/volume defects, which are only present in fine NIF powder. Since the other, coarser powders still have rather large surface areas and large amounts of surface defects (see, e.g., micrographs in [44]), the present 20–50 μm powder can differ (due to its smaller size and more intense grinding) only by these internal defects. As a consequence, the small crystallization pre-peak at ~37–43 °C should dominantly correspond to the manifestation of the GC growth mechanism, which initiates from exactly these types of active centers (internal micro-cracks, etc.).

Worth noting is also the sharp melting peak with the onset at 168.2 °C shown in Figure 1A, which indicates the amorphous-to-crystalline transformation leading to an exclusive formation of the high-temperature α_p_ polymorphic phase. In the context of the NIF crystallization data presented in [44,54,55,56,57], the absence of the endothermic features at 75, 137, and 157 °C rules out the β_p_ → β_p_′ polymorphic recrystallization or the formation of the γ′ and δ_p_ polymorphs, respectively. In Figure 1B, the evolution of the glass transition effect with *q^+^* is shown; only the data for higher *q^+^* are depicted, where the endothermic step-like change is not masked by the crystallization pre-peak (corresponding to the combined GC and enhanced surface growth modes). As can be seen, the relaxation peak (endothermic characteristics of the glass transition) seems to converge to approx. 35 °C with decreasing *q^+^*. Note that the decreasing magnitude of the relaxation peak (depth of the endothermic effect) with decreasing *q^+^* is not only a consequence of the above-mentioned masking but also a result of the structural relaxation phenomena generally producing a smaller “overshoot” at low *q^+^* [26,28].

**Figure 2:**
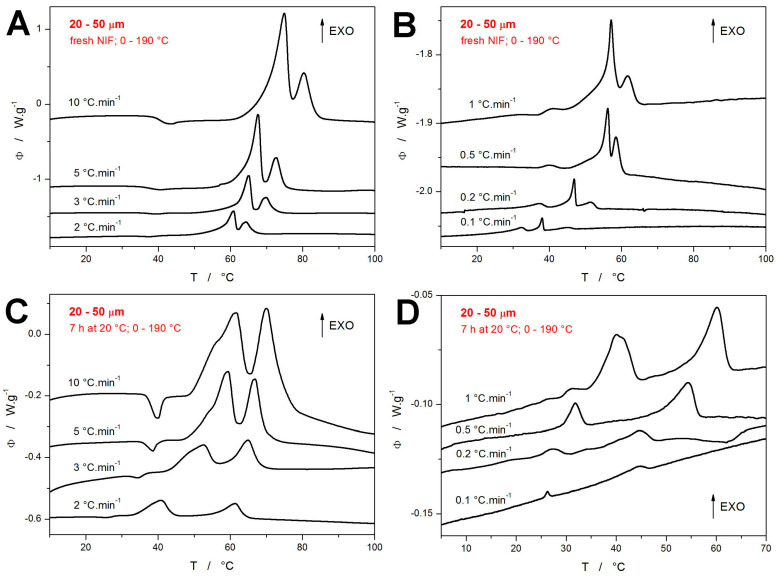
(**A**,**B**) DSC curves obtained at different q^+^ for the freshly prepared NIF powder. (**C**,**D**) DSC curves obtained at different *q^+^* for the NIF powders annealed for 7 h at 20 °C. Exothermic effects evolve in upward direction in all graphs.

The crystallization data obtained for the present 20–50 μm NIF powders are shown in Figure 2. The top row of graphs (Figure 2A,B) depict the DSC curves measured for the freshly prepared powders heated from 0 to 190 °C (the graphs are zoomed-in on the crystallization regions). The bottom row of graphs (Figure 2C,D) then shows the DSC measurements for the powders annealed at 20 °C for 7 h prior to the non-isothermal measurement, which was again performed in the 0–190 °C range. Before commenting on the particular graphs, it is worth reminding that each DSC curve corresponds to a measurement performed for a newly prepared NIF powder. From this point of view, the very good reproducibility of the crystallization features and trends is immediately apparent in the figures. In the case of the quickly heated fresh NIF powders (Figure 2A), the typical crystallization double-peak occurs, which (according to the temperature-resolved Raman spectroscopy measurements [44]) corresponds to the simultaneous formation of the α_p_ and β_p_ polymorphic phases (during the first crystallization peak) and the consequent β_p_ → α_p_ recrystallization (during the second crystallization peak). As *q^+^* decreases, the first crystallization peak starts to exhibit a more prolonged onset tail (indicating the α_p_ formation), and at *q^+^* ≤ 1 °C·min^−1^, the crystallization pre-peak starts to occur, increase in size, and decrease in temperature.

On the other hand, the non-isothermal crystallization following the 7 h annealing at 20 °C (during which the GC growth was allowed to proceed) occurs at significantly lower temperatures compared to the freshly prepared NIF samples—see Figure 2C,D. At higher *q^+^*, the crystallization starts to overlap with the glass transition signal already at 3 °C·min^−1^, and the endothermic relaxation peak practically disappears (is fully masked by the exothermic crystal growth) at *q^+^* ≤ 2 °C·min^−1^. The crystallization complexity monitored at higher *q^+^* appears to be rather similar to that of the fresh samples—a similar double-peak shifting to lower *T* with decreasing *q^+^*. However, for *q^+^* ≤ 2 °C·min^−1^, the two exothermic peaks start to separate, and the first (low-*T* one) appears to replace/imitate what the crystallization pre-peak represented in the case of the fresh NIF samples. For *q^+^* ≤ 0.5 °C·min^−1^, the first crystallization peak is already positioned at *T* < *T_g_*, unambiguously corresponding to the macroscopic manifestation of the diffusionless GC growth. It is also noteworthy that the crystallization signals obtained at low *q^+^* for the annealed NIF powder are significantly lower in magnitude compared to the similar signals measured for the fresh powder. This will be further commented on later in this section.

**Figure 3:**
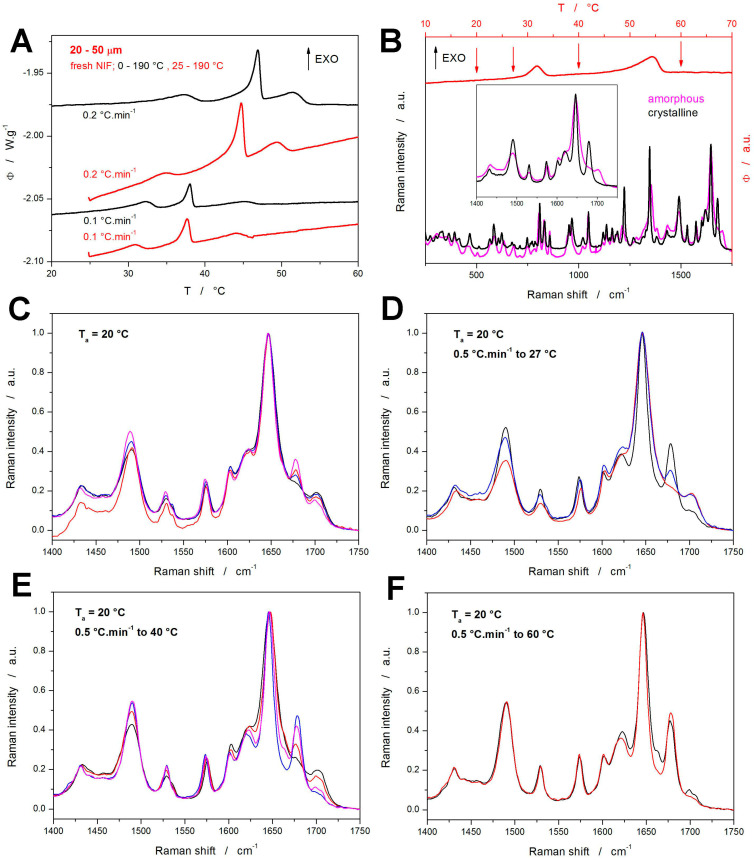
(**A**) Comparison of the DSC curves obtained non-isothermally at 0.1 and 0.2 °C·min^−1^ for the freshly prepared NIF powder. The measurement started either at 0 °C (black data) or 25 °C (red data). (**B**) DSC curve measured for the annealed NIF powder at 0.5 °C·min^−1^ (red data, top and right axes). The bottom part of the graph shows the Raman spectra collected for the fully amorphous and fully crystalline NIF samples; the inset shows the region used to best distinguish between the different NIF forms (amorphous, and α_p_ and β_p_ polymorphs). (**C**–**F**) Raman spectra collected at different temperatures indicated by arrows in Figure 1B; for each temperature, several spectra (represented by different colors in the graphs) were collected at different spots on the sample surface.

All DSC measurements reported in Figure 1 and Figure 2 were started at 0 °C, which gave several hours of exposition to the sub-*T_g_* temperatures for the samples heated at very low *q^+^* (≤0.5 °C·min^−1^). To explore the supposed impact of this effect, two testing heating scans were performed for the fresh NIF samples starting at 25 °C (instead of the previously used 0 °C)—see Figure 3A. In such cases, one would expect the crystallization peaks to occur at higher temperatures due to the shorter time allowed for the material nucleation, which would lead to a general slow-down of the overall macroscopic crystal growth. Surprisingly, the crystallization phenomena occur during these test scans at slightly lower temperatures compared to the cases with the NIF powders being heated from 0 °C. Since the temperature shift is very small, it can be neglected for the purposes of the kinetic calculations (this will be further discussed in Section 4) Regarding the possible explanations for this finding, any atmosphere-induced (oxidative) passivating alteration of the sample surface (associated with the prolonged exposition to the surroundings) can probably be ruled out because the volume-located GC growth (represented by the crystallization pre-peak) is also affected. Since the crystallization enthalpies (areas of the crystallization peaks) are rather similar, the possibility of a certain portion of the nuclei being depleted by the slow undetected growth during the slow heating from 0 to 25 °C can also be ruled out. Hence, the most probable explanation appears to be associated with the increased internal stresses caused by thermal contraction at low temperatures, where these stresses hinder the nucleation processes. An alternative explanation might involve a prolonged self-healing of the active crystallization centers at low temperatures (without which the centers would be open to the nucleation and growth), but this is quite implausible since the self-healing would certainly require higher mobility than the nucleation and growth processes.

The nature of the observed crystallization peaks was revealed by Raman microscopy. For the fresh samples, an akin study was performed already in [44], where the temperature-resolved measurements have shown that the crystallization double peak is initiated by the formation of the α_p_ polymorph (this is valid for both the sub-*T_g_* GC growth and the onset of the first main crystallization peak). During the later evolution of the first crystallization peak, the formation of the α_p_ polymorph is accompanied by the minor formation of the β_p_ form. The β_p_ polymorph may, under certain conditions, undergo a marked endothermic β_p_ → β’_p_ transformation (not seen in the present case of the 20–50 μm powder). During the second crystallization peak, the β_p_ and β’_p_ polymorphs recrystallize into the α_p_ form; further formation of the α_p_ polymorph is also expected at previously elided sites (that are energetically more favorable with increasing temperature) [44]. In the present study, in addition to the verification/reproduction of the results reported in [44], additional Raman measurements were performed for the annealed NIF powders (7 h at 20 °C) that were consequently heated at 0.5 °C·min^−1^ from 0 to 190 °C.

The DSC curve obtained for the fresh NIF powder at 0.5 °C·min^−1^ is shown in Figure 3B—the arrows indicate the temperatures at which the Raman measurements were performed. In Figure 3B, the fully amorphous and fully crystalline Raman spectra for NIF are also shown; the inset depicts the spectral region well suited for distinguishing between the amorphous and crystalline forms. The major Raman bands observed for NIF can be attributed as follows [58]: 810 and 836 cm^−1^~out of plane C-H ring vibration, 967 cm^−1^~dihydropyridine ring (possibly), 1048 cm^−1^~1,2-substituted ring, 1224 cm^−1^~C-C-O ester bond vibration, 1348 cm^−1^~symmetric stretching vibration of NO_2_, 1492 cm^−1^~C=C bond in aromatic circle, 1532 cm^−1^~asymmetric stretching vibration of NO_2_, 1575 cm^−1^~out of plane N-H scission, 1602 cm^−1^~C=C bond in aromatic circle, 1646 cm^−1^~stretching C=C vibration, and 1679 cm^−1^~C=O stretching vibration. The β_p_ polymorph then does not exhibit the 1679 cm^−1^ band but shows two new weak bands at 1664 and 1703 cm^−1^; the position of the main band at 1646 cm^−1^ is also shifted to ~1650 cm^−1^.

The Raman spectra corresponding to the four temperatures indicated on the DSC curve in Figure 3B (20, 27, 40, and 60 °C) are shown in Figure 3C,F (each graph corresponds to one temperature), the spectra were collected at multiple spots to comprise the span of the crystallinity degree. In each graph, only a few selected spectra are shown to demonstrate the maximum observed differences. Starting with Figure 3C, which depicts the Raman spectra collected just after the 7 h annealing at 20 °C, fully amorphous as well as slightly crystalline spots were found—note that the degree of crystallinity (amount of the α_p_ polymorph being present) is reflected by the height of the band at 1675 cm^−1^. By heating to 27 °C (just below the first crystallization peak), the span of the 1675 cm^−1^ band intensities slightly increased, indicating the slow crystal growth continuing even below the first dominant DSC effect. The heating to 40 °C (above the first crystallization peak) resulted in all spectra exhibiting at least a small amount of crystallinity, with occasional evidence for the traces of the β_p_ polymorphic phase (indicated by a very small band at 1663 cm^−1^). Nonetheless, at this stage, the dominantly encountered Raman signal was that corresponding only to a very small amount of the crystalline phase. This seems to be rather convincing evidence of the first crystallization peak dominantly representing the sub-*T_g_* GC growth, which is volume-located, proceeding along the internal micro-cracks.

Note that at 40 °C, more than 30% of the NIF powder was already crystalline (based on the DSC data), and thus the lack of the crystalline Raman signal may reflect the fact that the Raman microscopy is surface-sensitive, monitoring the surface layer of ~5 μm (the measurements were performed using the ×10 objective with declared spot size of 3.1 μm being focused on the surface of the grains). If, as is argued, the first crystallization peak dominantly corresponded to the volume-located GC growth, the surface of the NIF powder grains would still be largely amorphous, as indeed recorded by the Raman spectroscopy. By heating the samples to 60 °C, i.e., above the second crystallization peak, only the spectra corresponding to fully crystalline NIF were recorded; interestingly, the β_p_ phase Raman bands were still occasionally recorded, indicating that the β_p_ → α_p_ recrystallization did not take place.

**Figure 4:**
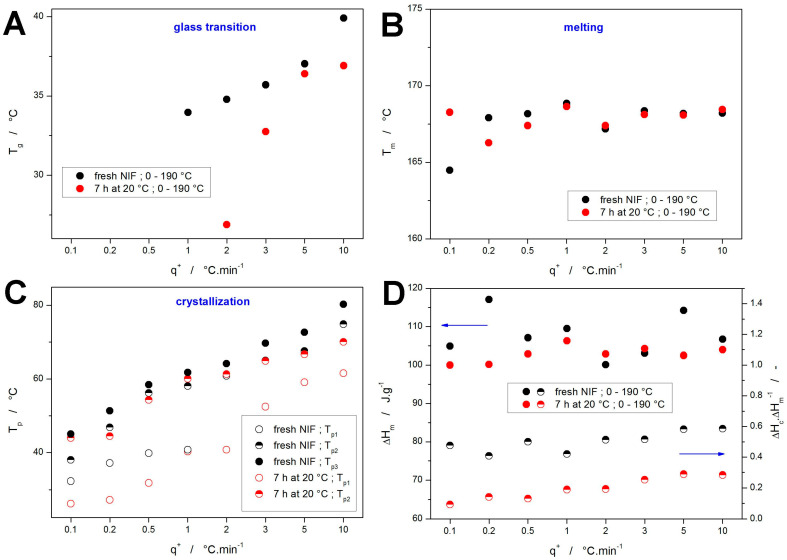
Evolution of *T_g_* (**A**), *T_m_* (**B**), and *T_p_*s (**C**) with *q^+^* for the freshly prepared and annealed (7 h at 20 °C) NIF powders. (**D**) The top half of the graph (+left axis) shows evolution of Δ*H_m_* with *q^+^* for the freshly prepared and annealed NIF powders. The bottom half (+right axis) of the graph depicts the Δ*H_c_*/Δ*H_m_* quantity.

The base quantification of the DSC data depicted in Figure 1 and Figure 2 is given in Figure 4. Starting with the glass transition temperature (evaluated as the half-height midpoint), the data in Figure 4A show the expected slow decrease of *T_g_* with decreasing *q^+^*—note that for the low *q^+^*, the glass transition effect was fully masked by the overlapping exothermic crystallization peak. In the case of the NIF powder annealed at 20 °C for 7 h, the *T_g_* evaluation at 2 and partially also 3 °C·min^−1^ was performed only based on the onset temperature of the partially masked effect—hence the significantly lower *T_g_* values. However, despite this change in the evaluation methodology, the *T_g_* onset still appears to be 3–4 °C below what would be expected, which may suggest that the presence of the crystalline phase may significantly later the glass transition dynamics—the similar effect was indeed already observed in the structural relaxation study of partially crystallized indomethacin powders [59]. On the contrary, the melting temperature *T_m_* (evaluated as the extrapolated onset) shows a marked invariance with experimental conditions, indicating the dominantly melting phase being the α_p_ polymorph—see Figure 4B. The only exception seems to be the fresh NIF powder crystallized at 0.1 °C·min^−1^, where the onset of the melting peak indicates a significant presence of the β_p_ crystalline phase.

Regarding the crystallization processes, they have been characterized by the temperatures corresponding to the maxima of the particular crystallization peaks *T_p_*—their evolution with *q^+^* is shown in Figure 4C. Here, the main conclusion seems to be the correspondence between the second (high-*T*) crystallization peak of the annealed NIF samples with the double peak of the fresh NIF samples. Accordingly, the first (low-*T*) crystallization peak recorded for the annealed NIF powders evidently represents the GC growth (or a continuation thereof), as indicated by the joint shifted trend in the *T_p_*-*q^+^* dependences. The probably most important information is then contained in Figure 4D, where the top part (filled points and left axis) shows the melting enthalpies Δ*H_m_*, and the bottom part (half-filled points and the right axis) depicts the ratio between the crystallization and melting enthalpies Δ*H_c_*/Δ*H_m_*—this ratio essentially represents a normalized quantity indicating the portion of the crystalline phase being formed during the crystallization peaks (as opposed to the crystal growth proceeding during the 7 h annealing or during the consequent heating from 0 to 30 °C). The melting enthalpies are fairly uniform, with the deviations possibly caused by inaccuracies of measurements or evaluations, which indicates that a similar amount of crystalline phase is always formed within the sample. Considering the other data reported in this study so far, this finding indicates that the NIF powders are fully crystallized under all explored conditions. The Δ*H_c_*/Δ*H_m_* ratios then indicate that a large portion (approx. 30–50%) of the material was already crystallized during the 7 h annealing at 20 °C. Note that the exact percentage is quite difficult to determine since Kirchhoff’s law obviously also affects the measured Δ*H_c_* values (as evidenced by the Δ*H_c_*/Δ*H_m_* ≈ 0.6 ratio obtained for the fresh, quickly heated NIF powder). Nonetheless, this further supports the idea of a large portion of the initial crystalline phase being formed via the GC growth within the NIF powder grains (as indicated by the almost amorphous Raman spectra shown in Figure 3C). The further decrease in the crystallization enthalpy with decreasing *q^+^* for the annealed NIF samples indicates that the slow (DSC-undetected) crystal growth (most probably of a similar nature as during the long-term annealing) proceeds also during the initial heating within the heating scan applied after the annealing isotherm—although a certain impact of the Kirchhoff’s law lessens the seemingly large decrease of Δ*H_c_* within this period. As these effects are suppressed/negligible for the Δ*H_c_*/Δ*H_m_* data obtained at 10 °C·min^−1^, these data will be used as a benchmark for the kinetics calculations introduced in Section 3. The main purpose of these calculations/predictions will be to test the accuracy of the determination of the degree of GC growth crystallinity and kinetics from the macroscopic DSC data.

## 3. Discussion

The main asset of the detailed thermo-kinetic studies based on a series of heating scans performed at different *q^+^* is the possibility to model the corresponding kinetics and make accurate kinetic predictions. The crystallization from a glassy state (sometimes also denoted as “cold crystallization”) is standardly described in terms of the solid-state kinetic equation [60]:

(1) $$\Phi =∆H_{c}\cdot A\cdot e^{-E/RT}\cdot f(\alpha )$$

where *Φ* is the DSC heat flow signal, Δ*H_c_* is the crystallization enthalpy, *A* is the pre-exponential constant, *E* is the activation energy of crystallization, *R* is the universal gas constant, and *f*(*α*) is a function defining the shape of the crystallization peak as a dependence of the degree of conversion *α.* One of the most flexible solid-state kinetic models used for the crystallization processes is the semi-empirical autocatalytic Šesták–Berggren [61] (AC) model:

(2) $${f(\alpha )}_{AC}=\alpha^{M}{\left(1-\alpha \right)}^{N}$$

where *M* and *N* are the exponents of the AC kinetic model. Before applying this equation, the thermo-kinetic background (DSC baseline) needs to be subtracted from the experimental data. In this regard, the physically meaningful tangential area-proportional baseline [61] was used:

(3) $$B\left(T\right)=\left(1-\alpha \left(T\right)\right)\cdot \left(z_{0,r}+z_{1,r}\cdot T\right)+\alpha \left(T\right)\cdot \left(z_{0,p}+z_{1,p}\cdot \left(T_{f}-T\right)\right)$$

where *B*(*T*) is the temperature dependence of the baseline curve, *α* is the degree of conversion, *z*_0,*r*_ and *z*_1,*r*_ are the coefficients characterizing the tangent going through the starting point (in the reactants area), *z*_0,*p*_ and *z*_1,*p*_ are the coefficients characterizing the tangent going through the endpoint (in the products area), and *T_f_* is the endpoint temperature.

Since the NIF crystallization signals are clearly complex, consisting of multiple overlapping peaks, the multivariate kinetic analysis (MKA) expressed by the following set of equations had to be used to set the framework for the non-linear optimization procedure (curve-fitting) based on the Levenberg–Marquardt algorithm:

(4) $$RSS=\sum_{j=1}^{n}\sum_{k=First_{j}i}^{Last_{j}}w_{j,k}{\left(Y\exp_{j,k}-Ycal_{j,k}\right)}^{2}$$

(5) $$\frac{d\alpha_{x}}{dt}=A_{x}\cdot exp\left(-\frac{E_{x}}{RT}\right)\cdot \alpha_{x}^{M}\cdot {\left(1-\alpha_{x}\right)}^{N}$$

(6) $$1=\sum_{x=1}^{n}I_{x}\cdot \alpha =\sum_{x=1}^{n}\alpha_{x}$$

where *RSS* is the sum of squared residue, *n* is a number of measurements, *j* is an index of the given measurement, *First_j_* is the index of the first point of the given curve, *Last_j_* is the index of the last point of the given curve, *Yexp_j,k_* is the experimental value of the point *k* of curve *j*, *Ycal_j,k_* is the calculated value of the point *k* of curve *j* (calculated in accordance with Equation (4)), and *w_j_* is a weighting factor for curve *j* (will be set to unity, as explained later). The index “x” in Equations (5) and (6) indicates the given sub-process, and “*n*” is the number of sub-processes (3–5 in the present case), while *I_x_* represents a proportional representation for each sub-process. As the classical MKA method fits all measured DSC curves simultaneously, it is very sensitive to any data distortions causing the shift in the kinetic (crystallization) peaks along the temperature axis. The method is often unusable for the crystallization processes, which tend to have lower reproducibility due to their complexity [62]. The MKA method is also inapplicable to the data exhibiting temperature-dependent kinetics, which is again a common case in crystallization processes. For this reason, a modification of the MKA method was developed under the designation “single-curve multivariate kinetic analysis” (sc-MKA) [63]. This approach utilizes the separate (ideally independent) determination of *E_x_* values, which are then fixed for the non-linear optimization. With the fixed *E_x_* values, the *E_x_*-*A_x_* correlation is no longer a problem, and each DSC curve (obtained at different *q^+^*) can be optimized individually, providing its own set of kinetic parameters. In this way, the trends evolving in the kinetic parameters with changing *q^+^* suitably describe the temperature-dependent kinetics. In addition, the inter- or extrapolations can be performed much more accurately due to the possibility of selecting the corresponding temperature range (characterized by *q^+^*).

**Figure 5:**
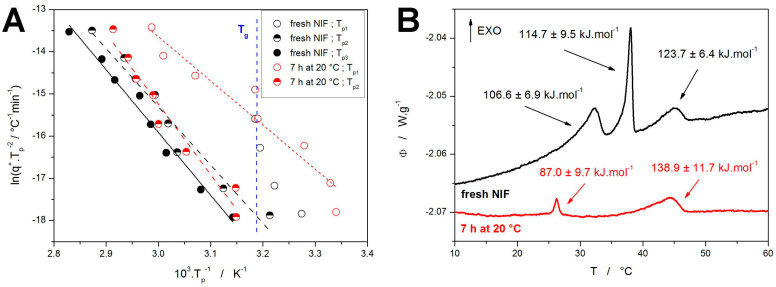
(**A**) Kissinger dependences calculated from the T_p_ data shown in Figure 4C (points); black and red lines correspond to the linear fits of these dependences. Vertical blue line indicates the position of *T_g_*. (**B**) DSC curves obtained for the two types of NIF samples at 0.1 °C·min^−1^; activation energies determined by the Kissinger method are attributed to the particular crystallization sub-processes.

As mentioned above, the first step in the application of the sc-MKA method is the determination of *E_x_* values (activation energies for the individual sub-processes). The most reliable and robust way is, in this regard, the utilization of the Kissinger method [64]:

(7) $$ln\left(\frac{q^{+}}{T_{p}^{2}}\right)=-\frac{E}{RT_{p}}+const.$$

where *T_p_* is the temperature corresponding to the maximum of the crystallization peak (as shown in Figure 4C). The Kissinger dependences calculated for the present crystallization data are shown in Figure 5A, together with the estimated averaged position of *T_g_*. A relatively good linearity of the Kissinger dependences proves the very good reproducibility of the data (considering that each point within the given dependence was obtained for a newly prepared NIF powder). Out of the two types of measurements, the worse reproducibility/linearity is obtained for the samples annealed for 7 h at 20 °C, where the annealing magnifies the deviations in the nucleation intensity during the quench-in and sample-processing periods. Interesting behavior is observed for the crystallization peak corresponding to the GC growth in fresh NIF samples, where its position coincides with and limits to the *T_g_* value. This can be understood from the perspective of the inherent nature of the GC growth, which ceases above *T_g_* [49]. In Figure 5B, the activation energies calculated from the Kissinger dependences are attributed to the particular crystallization processes—as depicted for the measurements performed at 0.1 °C·min^−1^. In addition to the Kissinger method, the more complex isoconversional methods were applied to determine/estimate the dependence of *E* on *α* during the course of the overall crystallization process. The results are shown in the Supplementary Material—due to the complexity of the NIF crystallization behavior, and due to the inability of the isoconversional methods [65,66] to reflect the temperature-dependent kinetics [67,68], no added benefit was obtained by application of these methods.

**Figure 6:**
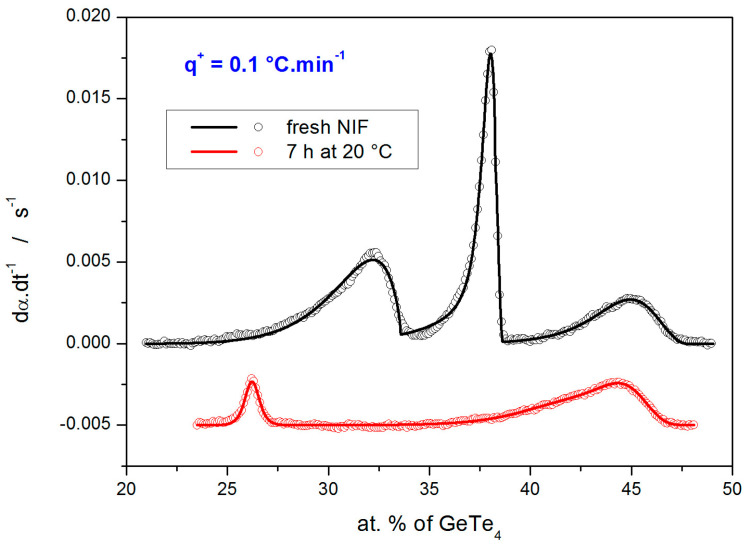
DSC curves obtained for the two types of NIF samples at 0.1 °C·min^−1^ (points) fit by the complex crystallization models based on Equations (5) and (6) (lines).

Examples of the quality of the sc-MKA fits to the experimental data (DSC curves obtained at 0.1 °C·min^−1^) are shown in Figure 6. The overall averaged correlation coefficients associated with all sc-MKA fits were *r* = 0.9990 and 0.9973 for the fresh and annealed NIF samples, respectively.

**Figure 7:**
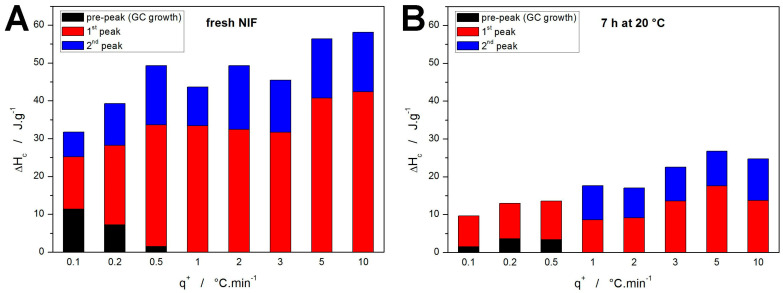
Crystallization enthalpies obtained by means of sc-MKA method for the fresh (**A**) and annealed (**B**) NIF samples. The attribution of the particular sub-processes for the annealed NIF samples is discussed at length in the text.

The complete sets of kinetic parameters for the two types of samples are listed in Table 1 and Table 2. The most important data (apart from the *E_x_* values) are the proportional crystallization enthalpies indicating the representation of the individual crystallization sub-processes—these data are shown in Figure 7. Whereas for the fresh NIF samples, the assignment of the particular sub-processes is straightforward, it is somewhat questionable for the annealed samples (Figure 7B). The present assignment corresponds to the temperature ranges, where for the *q^+^* ≤ 0.5 °C·min^−1^, the first DSC-recorded peak occurs below *T_g_* (hence the “shift” of the colors). Despite the assignment, it is clear that over 50% of the initially amorphous NIF material crystallizes during the 7 h annealing. Furthermore, judging on the evolution of the kinetic parameters and Δ*H_c,x_* values, the intensity and proportional representation of the GC growth (associated with the crystallization pre-peak) increases with decreasing *q^+^*. This is a fundamental finding that will play a crucial role in the kinetic predictions.

**Figure 8:**
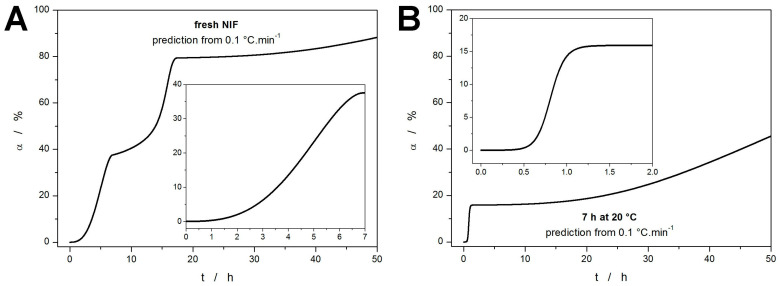
Kinetic predictions of the degree of amorphous-to-crystalline conversion for the fresh (**A**) and annealed (**B**) NIF samples during the isotherm at 20 °C. The predictions are based on the kinetic parameters obtained for the DSC measurements performed at 0.1 °C·min^−1^. The insets show the data zoomed-in on the initial part of the graph.

The ultimate goal of the kinetic calculations and modeling is the kinetic predictions. In the present case, the data obtained for the annealed NIF sample (7 h at 20 °C) heated at 10 °C·min^−1^ can be used as a reference value for the predictions made from the kinetic parameters obtained for the fresh NIF sample. Note that the measurement performed at 10 °C·min^−1^ is only negligibly influenced by the additional sub-*T_g_* formation of the crystalline phase (undetected by the DSC) during the heating from 0 °C to *T_p1_*, hence its selection as a reference value. If the uncertainty of the Δ*H_c_* is considered (by averaging over the measurements performed at 5 and 10 °C·min^−1^), the 7 h annealing at 20 °C resulted in ~50–60% crystallinity. To achieve the most accurate results, the sc-MKA results for the lowest *q^+^* need to be used in order to best approximate the isothermal annealing at a lower temperature (extrapolation to *q^+^* = 0 °C·min^−1^). The kinetic prediction calculated using the parameters obtained for the fresh NIF powder heated at 0.1 °C·min^−1^ is shown in Figure 8A. The theoretically simulated curve shows three distinct steps corresponding to the three crystallization sub-processes. According to this prediction, approx. 35–40% crystallinity is achieved after 7 h annealing at 20 °C—the experimentally obtained value was 50–60%. This discrepancy indicates that the kinetic parameters obtained at *q^+^* = 0.1 °C·min^−1^ should have been further extrapolated to lower *q^+^*. This is particularly relevant with regard to the sharply increasing portion of Δ*H_c,1_* corresponding to the crystallization pre-peak (GC growth) in Figure 7A. Such correction would obviously increase the predicted degree of crystallinity associated with the formation of the GC-growth-induced phase, better approximating the reality.

A further indication of the necessity to extrapolate the kinetic parameters obtained even at *q^+^* as low as 0.1 °C·min^−1^ is demonstrated in Figure 8B, where the initial step indicates achieving an additional~15% of crystallinity within 1 h of annealing at 20 °C—in this case, the starting material was the NIF powder already annealed for 7 h at 20 °C. A clear shortening of the crystallization time from 15 h to 1.5 h (compared to the data shown in Figure 8A) is apparent. According to the author’s experience with a large variety of crystallizing materials, such necessity for major extrapolations, even from the very low heating rates, appears to be specific for the crystallization of the low-molecular glasses (typically amorphous drugs) that exhibit a strong GC growth. Despite the present underestimation of the crystal growth rate, the overall concept of direct monitoring of the GC growth by DSC is hereby verified. Note that for pharmaceutical purposes, the accuracy of the kinetic prediction demonstrated in Figure 8A is still extremely good and well above the present state of knowledge in the field.

Regarding further improvements of the mathematic methodology for the kinetic predictions, the linearity of the Kissinger plots evokes the idea of linear transposition of the kinetic parameters with the reciprocal temperature from *T_p_* of the measurement performed at lowest *q^+^* to *T_a_*. The potential and validity of such an idea remains to be seen.

## 4. Materials and Methods

The melt–quench technique was used to prepare the amorphous NIF from its α_p_ polymorphic form (Sigma-Aldrich, Prague, Czech Republic: 99.5% purity). In particular, the fine crystalline powder was placed into a glass vial, melted in an oil bath tempered to 175 °C, and quenched (still within the vial) in cold water. The glassy NIF ingot (volume of ~70 μL) was then broken, gently ground with an agate mortar and pestle to a fine powder, and sieved through the sieves (Retsch/Verkon, Prague, Czech Republic) with defined mesh to obtain a few mg of the 20–50 μm amorphous NIF powder. Since NIF is a light-sensitive compound [69,70], the majority of the above-described procedures were performed in the dark, and the exposure to light was minimized to ~5–10 min. The prepared amorphous NIF powder was immediately hermetically sealed into a low-mass Al DSC pan; the sample masses were ~2–4 mg (accurately weighted to 0.01 mg)—hence, no relevant amount of moisture could be adsorbed onto the powder. The sample was then again immediately subject to the selected DSC temperature program.

All steps described in the previous paragraph were performed and repeated for each sample, i.e., each sample was prepared individually, including the melt–quenching, grinding, and sieving. In this way, not only was the reproducibility of the overall sample preparation method extensively tested, but any potential influence of the storage conditions was also nullified. Note that during our previous studies [44] of the NIF thermal characteristics, even the storage in dry, dark, and cold (0 °C) conditions appeared to alter the nucleation (and consequently crystallization) behavior, especially in the case of very finely ground powders—hence the present treatment.

The DSC measurements were performed using the differential scanning calorimeter (DSC) Q2000 (TA Instruments, New Castle, DE, USA) equipped with an autosampler, an RCS90 cooling accessory, and T-zero technology. The DSC calibration was performed with the In, Zn, and H_2_O standards. The DSC measurements were performed in the hermetically sealed low-mass Al pans (i.e., in a static air atmosphere); the N_2_ flow of 50 mL·min^−1^ was still preserved in the DSC cell to ensure the stabilization of the thermal gradients throughout the measurement. Three types of DSC measurements were performed to cover different alternatives of the GC growth manifestation. The first fundamental type of measurement was the simple heating scan of a freshly prepared sample in the 0–190 °C temperature range. The applied heating rates were *q^+^* = 0.1, 0.2, 0.5, 1, 2, 3, 5, and 10 °C·min^−1^; each heating rate corresponds to a separate measurement with freshly prepared NIF sample. The second type of measurement was essentially similar but preceded with a 7 h isotherm at 20 °C (the isotherm was also realized as a part of the DSC measurement to guarantee the accuracy of the thermal treatment.) Again, the heating rates consequently applied in the 0–190 °C were in the 0.1–10 °C·min^−1^ range. The third type of DSC measurement was identical to the first type of measurement, but the explored temperature range was reduced to 25–190 °C so that the time given for the nucleation and/or local microscopic growth below *T_g_* is minimized. The third type of the DSC measurement was realized only for the lowest *q^+^* (0.1, 0.2 °C·min^−1^), for which the heating within the 0–25 °C interval still represents a significant time period allowed for the GC growth to potentially proceed.

Confirmation of the amorphous character of the freshly prepared NIF powders and the identification of the polymorphic phase emerging during the GC growth were performed by means of Raman microscopy. The DXR2 Raman microscope (Nicolet, Thermo Fisher Scientific, Prague, Czech Republic), equipped with a 785 nm excitation diode laser (30 mW, laser spot size of 3.1 μm) and CCD detector, was utilized under the following conditions: a 10 mW laser power on the sample, 10 s duration of a single scan, and 30 scans summed in one spectrum.

## 5. Conclusions

Different crystallization modes occurring in the finely ground amorphous (20–50 μm) NIF powder were studied by means of DSC and Raman microscopy. At very low heating rates (0.1–0.5 °C·min^−1^), a macroscopic manifestation of the sub-*T_g_* GC growth could be directly observed in the form of an exothermic peak recorded during the DSC measurements of the freshly prepared NIF powder. The position of the DSC GC growth peak was indeed below *T_g_*, and the process ceased as the glass transition occurred. The activation energy of the DSC-monitored GC growth was ~106 kJ·mol^−1^, i.e., significantly lower compared to the other crystallization modes occurring above *T_g_*. In addition to the DSC measurements of the freshly prepared NIF samples, a similar range of heating rates was also applied to the NIF powders previously annealed for 7 h at 20 °C (i.e., below *T_g_*), which already achieved~50–60% crystallinity during the annealing. The annealed samples exhibited very little crystallinity on the surface (revealed by the confocal Raman microscopy), which supports the idea of a majority of the α_p_ polymorphic crystalline phase being formed within the amorphous NIF grains, i.e., via the GC growth mechanism. In the case of the consequent non-isothermal measurements of the annealed samples, the pure GC growth apparently disappeared (probably due to the saturation of the active volume-located crystallization centers, i.e., internal micro-cracks) but was replaced by an akin sub-*T_g_* growth mode, which probably represents a secondary growth from the crystals formed during the GC growth.

All DSC data were modeled in terms of the solid-state kinetic equation paired with the autocatalytic model; the kinetic complexity was described via reaction mechanism based on the overlap of 3–4 independent processes. Owing to the sc-MKA methodology, clear trends in the kinetic description could be identified with decreasing *q^+^*, which indeed confirmed an increasing portion of the GC growth-induced crystallinity with increased time spent during the heating below *T_g_*. The kinetic description of the DSC data were consequently used to make a kinetic prediction based on the temperature program identical to the experimentally performed annealing (7 h at 20 °C). The kinetic model slightly underestimated the true extent of the GC growth, predicting the crystallinity being 35–40% after 7 h (such accuracy is still extremely good in comparison with the nowadays standard kinetic approaches). The discrepancy indicates the necessity for extrapolation of the kinetic trends toward the true temperatures of the experiment—this should be the main direction of the research focused on modern kinetic analysis.

The most important conclusion is, however, that the GC growth in amorphous drugs can indeed be reproducibly monitored by the DSC technique, which allows for the investigation of its consequences in real-life dosage forms (usually utilizing finely powdered APIs). Moreover, the kinetic modeling and associated predictions of the GC growth manifestation can be made with well-sufficient accuracy for the needs of modern pharmaceutical technology.

## Figures and Tables

**Table 1:** Kinetic parameters obtained by sc-MKA for the fresh NIF samples. The sub-processes can be identified according to the *E_x_* values shown in Figure 5B; certain kinetic peaks required two sub-processes to be accurately fit. *A_x_* are in “s^−1^”, *E_x_* are in “kJ·mol^−1^”, Δ*H_x_* are in “J·g^−1^”.

*q* ^+^	0.1	0.2	0.5	1	2	3	5	10
log(*A*_1_)	16.98	16.69	16.47	16.81	16.71	16.54	16.71	16.53
*E*_1_	114.70	114.70	114.70	114.70	114.70	114.70	114.70	114.70
*N*_1_	0.62	0.58	0.69	1.51	0.68	0.66	0.76	0.85
*M*_1_	1.13	1.11	1.09	1.11	1.03	0.98	1.02	0.99
log(*A*_2_)	17.38	17.20	17.61	17.51	17.77	17.72	17.72	17.44
*E*_2_	123.70	123.70	123.70	123.70	123.70	123.70	123.70	123.70
*N*_2_	0.68	0.57	0.99	0.89	1.17	1.37	1.06	1.01
*M*_2_	0.81	0.76	1.02	0.91	0.96	1.01	0.98	0.91
log(*A*_3_)	16.13	15.87	15.78	15.92	16.13	15.92	16.13	15.99
*E*_3_	114.70	114.70	114.70	114.70	114.70	114.70	114.70	114.70
*N*_3_	0.12	0.16	0.38	0.34	0.37	0.32	0.40	0.43
*M*_3_	0.65	0.64	0.59	0.46	0.57	0.48	0.61	0.56
log(*A*_4_)	15.19	15.15	15.54					
*E*_4_	106.60	106.60	106.60					
*N*_4_	0.41	0.49	0.65					
*M*_4_	0.69	0.59	0.53					
Δ*H*_1_/Δ*H*	0.28	0.24	0.31	0.34	0.33	0.38	0.28	0.23
Δ*H*_2_/Δ*H*	0.21	0.28	0.32	0.23	0.34	0.30	0.28	0.27
Δ*H*_3_/Δ*H*	0.16	0.29	0.35	0.43	0.33	0.32	0.44	0.50
Δ*H_4_*/Δ*H*	0.36	0.18	0.03					
Δ*H*	31.73	39.24	49.25	43.61	49.32	45.45	56.35	58.12
*r*	0.9974	0.9983	0.9991	0.9993	0.9994	0.9993	0.9997	0.9997

**Table 2:** Kinetic parameters obtained by sc-MKA for the annealed NIF samples. The sub-processes can be identified according to the *E_x_* values shown in Figure 5B; certain kinetic peaks required two sub-processes to be accurately fit. *A_x_* are in “s^−1^”, *E_x_* are in “kJ·mol^−1^”, Δ*H_x_* are in “J·g^−1^”.

*q* ^+^	0.1	0.2	0.5	1	2	3	5	10
log(*A*_1_)	13.04	12.55	12.84	13.49	12.94	12.46	12.47	12.60
*E*_1_	87.00	87.00	87.00	87.00	87.00	87.00	87.00	87.00
*N*_1_	1.06	0.82	0.74	4.81	0.97	0.68	0.64	0.76
*M*_1_	1.00	0.77	0.78	1.24	0.89	0.82	0.87	0.84
log(*A*_2_)	20.04	20.18	20.08	19.64	19.89	19.99	20.31	20.27
*E*_2_	139.00	139.00	139.00	139.00	139.00	139.00	139.00	139.00
*N*_2_	0.75	0.77	0.77	0.51	0.70	0.91	1.19	1.52
*M*_2_	0.85	0.75	0.89	0.64	0.65	0.78	0.91	0.84
log(*A*_3_)	20.08	20.82	19.92	12.46	12.88	12.51	12.28	12.60
*E*_3_	139.00	139.00	139.00	87.00	87.00	87.00	87.00	87.00
*N*_3_	1.03	2.03	0.55	0.45	0.80	2.04	1.52	1.30
*M*_3_	0.72	0.95	0.61	0.65	0.71	0.71	0.62	0.63
Δ*H*_1_/Δ*H*	0.16	0.28	0.25	0.18	0.30	0.18	0.26	0.24
Δ*H*_2_/Δ*H*	0.46	0.56	0.47	0.51	0.46	0.40	0.34	0.45
Δ*H*_3_/Δ*H*	0.38	0.16	0.29	0.31	0.24	0.42	0.40	0.32
Δ*H*	9.68	12.94	13.56	17.64	17.02	22.52	26.71	24.73
*r*	0.9949	0.9955	0.9993	0.9937	0.9974	0.9993	0.9995	0.9992

## Data Availability

Raw data available at: 10.6084/m9.figshare.28738055.

## Supplementary Materials

The following supporting information can be downloaded at https://www.mdpi.com/article/10.3390/molecules30081679/s1, Isoconversional analyses of the crystallization in the freshly prepared and annealed NIF samples.
