# Supplementary material for: Calorimetric Monitoring of the Sub-Tg Crystal Growth in Molecular Glasses: The Case of Amorphous Nifedipine
Source: Molecules. 2025 Apr 9;30(8):1679. doi: 10.3390/molecules30081679 (PMC12029594; doi:10.3390/molecules30081679)
Supplement: Supplementary file 1 [file molecules-30-01679-s001.zip › molecules-3569134-supplementary.pdf]

Supplemental online material

The supplementary data (shown below) include the isoconversional analyses performed to determine the E-α dependences for the two types of the NIF samples—freshly prepared and annealed
for 7 h at 20 °C. The differential and integral methods are represented by the Friedman (Eq. S1) [S1] and Ozawa–Flynn–Wall (Eq. S2) [S2] methods, respectively:

$$\ln\left(\left[d\alpha/dt\right]_{\alpha}\right) = -\frac{E}{RT_{\alpha}} + const. \tag{S1}$$

$$\ln\left(q^{+}\right) = -1.0516\left(\frac{E}{RT_{\alpha}}\right) + const. \tag{S2}$$

where the index "α" denotes the arbitrarily selected degree of conversion (0.02, 0.05, 0.10, 0.15 … 0.90, 0.95, 0.98 in the present project). Considering the complexity of the reaction mechanisms (multiple overlapping crystallization sub-processes) paired with their temperature-dependent kinetics, the obtained E-α dependences do not provide any added information and are clearly surpassed by the simplicity, unambiguity, and interpretability of the Kissinger method.

[S1] Friedman, H.L. Kinetics of thermal degradation of char-forming plastics from thermogravimetry. Application to a phenolic plastic. J. Polym. Sci., Part C 1964, 6, pp. 183–195.
[S2] Flynn, J.H.; Wall, L.A. General treatment of the thermogravimetry of polymers. J. Res. Nat. Bur. Standards 1966, Part A 70, pp. 4.
